# Sex-Related Differences in Cardiovascular Risk in Adolescents with Overweight or Obesity

**DOI:** 10.31083/j.rcm2504141

**Published:** 2024-04-09

**Authors:** Procolo Di Bonito, Anna Di Sessa, Maria Rosaria Licenziati, Domenico Corica, Malgorzata Wasniewska, Emanuele Miraglia del Giudice, Anita Morandi, Claudio Maffeis, Maria Felicia Faienza, Enza Mozzillo, Valeria Calcaterra, Francesca Franco, Giulio Maltoni, Nicola Moio, Arcangelo Iannuzzi, Giuliana Valerio

**Affiliations:** ^1^Department of Internal Medicine, “S. Maria delle Grazie” Hospital, 80078 Pozzuoli, Italy; ^2^Department of Woman, Child and of General and Specialized Surgery, Università degli Studi della Campania “Luigi Vanvitelli", 80138 Napoli, Italy; ^3^Neuro-Endocrine Diseases and Obesity Unit, Department of Neurosciences, Santobono-Pausilipon Children's Hospital, 80129 Naples, Italy; ^4^Department of Human Pathology in Adulthood and Childhood, University of Messina, 98122 Messina, Italy; ^5^Department of Surgery, Dentistry, Pediatrics and Gynecology, Section of Pediatric Diabetes and Metabolism, University and Azienda Ospedaliera Universitaria Integrata of Verona, 37126 Verona, Italy; ^6^Pediatric Unit, Department of Precision and Regenerative Medicine and Ionian Area, University of Bari “Aldo Moro”, 70124 Bari, Italy; ^7^Department of Translational Medical Science, Section of Pediatrics, Regional Center of Pediatric Diabetes, University of Naples “Federico II”, 80131 Napoli, Italy; ^8^Pediatric Department, “V. Buzzi" Children's Hospital, 20154 Milano, Italy; ^9^Department of Internal Medicine, University of Pavia, 27100 Pavia, Italy; ^10^Pediatric Department, Azienda Sanitaria Universitaria del Friuli Centrale, Hospital of Udine, 33100 Udine, Italy; ^11^Pediatric Unit, IRCCS Azienda Ospedaliero-universitaria di Bologna, 40138 Bologna, Italy; ^12^Department of Cardiology, “S. Maria delle Grazie” Hospital, 80078 Pozzuoli, Italy; ^13^Department of Medicine and Medical Specialties, A. Cardarelli Hospital, 80131 Naples, Italy; ^14^Department of Medical, Movement and Wellbeing Sciences, University of Napoli “Parthenope”, 80133 Napoli, Italy

**Keywords:** adolescents, cardiometabolic risk, carotid intima media thickness, estimated glomerular filtration rate, fatty liver disease, left ventricular mass, sex, visceral adiposity

## Abstract

**Background:**

Pediatric obesity is closely associated with cardiometabolic comorbidities, but the role of sex in this relationship is less investigated. We aimed to evaluate sex-related differences on cardiometabolic risk factors and preclinical signs of target organ damage in adolescents with overweight/obesity (OW/OB).

**Methods:**

The main cross-sectional study included 988 adolescents (510 boys and 478 girls) with OW/OB aged 10–18 years. In all youths clinical and biochemical variables were evaluated and an abdominal echography was performed. Echocardiographic data for the assessment of left ventricular mass (LVM) and relative wall thickness (RWT) were available in an independent sample of 142 youths (67 boys and 75 girls), while echographic data of carotid intima media thickness (cIMT) were available in 107 youths (59 boys and 48 girls).

**Results:**

The three samples did not differ for age, body mass index, and sex distribution. In the main sample, boys showed higher waist-to-height ratio (WHtR) values (*p* < 0.0001) and fasting glucose levels (*p* = 0.002) than girls. Lower levels of estimates glomerular filtration rate (eGFR) were found in girls vs boys (*p* < 0.0001). No sex-related differences for prediabetes and hyperlipidemia were observed. A higher prevalence of WHtR ≥0.60 (57.3% vs 49.6%, *p* = 0.016) and fatty liver disease (FLD) (54.5% vs 38.3%, *p* < 0.0001) as well as a trend for high prevalence of hypertension (40.4 vs 34.7%, *p* = 0.06) were observed in boys vs girls. More, a higher prevalence of mild reduced eGFR (MReGFR) (<90 mL/min/1.73 $m^{2}$) was observed in girls vs boys (14.6% vs 9.6 %, *p* < 0.0001). In the sample with echocardiographic evaluation, boys showed higher levels of LVM (*p* = 0.046), and RWT (*p* = 0.003) than girls. Again, in the sample with carotid echography, boys showed higher levels of cIMT as compared to girls (*p* = 0.011).

**Conclusions:**

Adolescent boys with OW/OB showed higher risk of abdominal adiposity, FLD, and increased cardiac and vascular impairment than girls, whereas the latter had a higher risk of MReGFR. Risk stratification by sex for cardiometabolic risk factors or preclinical signs of target organ damage should be considered in youths with OW/OB.

## 1. Introduction

Notoriously, pediatric obesity represents an alarming phenomenon in industrialized countries from both a health and a socio-economic point of view. In addition, according to the World Health Organization, rate of children with obesity (OB) is expected to double by 2035 [1]. Noteworthy, this condition is closely associated with several comorbidities or preclinical signs of organ damage, such as prediabetes, hypertension, dyslipidemia, and fatty liver disease (FLD) [2]. This scenario is supposed to track into adulthood and produce serious long-term consequences on cardiometabolic health. Indeed, the early presence of obesity-related comorbidities may contribute to accelerate the risk of cardiovascular morbidity in adulthood [2].

The presence of cardiometabolic comorbidities has been largely studied in the pediatric population [3], but little is known about the influence of sex on this association. This aspect should not be overlooked in children, since it has been widely demonstrated that men are more frequently exposed to early morbidity and mortality for cardiovascular disease than females [4]. Given the well-documented protective role of oestrogens, this advantage is attenuated after menopause [5]. In addition, diseases associated to high cardiometabolic risk, such as type 2 diabetes [6] and FLD are more prevalent in males [7]. On the contrary, a higher risk of chronic kidney disease has been reported in females [8].

Given the high burden of cardiovascular risk in pediatric OB [9], it may be interesting to analyze in-depth whether and which kind of cardiovascular risk factors or preclinical signs of target organ damage are associated to a non-modifiable risk factor such as sex.

Based on these premises, we hypothesized that sex-related differences in cardiovascular risk might be present in adolescents with excess weight. Therefore, the aim of this study was to compare the traditional cardiovascular risk factors and preclinical signs of cardiac, vascular and renal impairment between adolescent boys and girls with overweight (OW) or OB.

## 2. Materials and Methods

This is a multicenter cross-sectional study that included 1562 youths with OW or OB. Participants were consecutively admitted to nine Italian endocrinology centers of the Pediatric Obesity Study Group within the Italian Society for Pediatric Endocrinology and Diabetology between 2016 and 2020 [10]. This study was conducted according to the principles expressed in the Declaration of Helsinki and approved by the Research Ethical Committee of University of Campania “Luigi Vanvitelli” (protocol code 834/2016). An informed consent was obtained from the parents of all participants before any procedure.

Exclusion criteria were: age <10 years (n = 561), genetic obesity or endocrine disorders, chronic use of medications, glucose levels in the diabetic range (n = 13). Finally, the records of 988 adolescents (510 boys and 478 girls), mean age 12.9 ± 1.8 years, were examined.

Two separate samples of 142 young people with OW or OB (67 boys and 75 girls) with echocardiographic evaluation performed in the Pozzuoli Hospital (Naples) [11], and 107 youths (59 boys and 48 girls) with carotid ultrasound performed in the Cardarelli Hospital of Naples [12] between 2003 and 2013, were included in the study.

### 2.1 Anthropometric, Clinical and Laboratory Evaluations

Anthropometric parameters were measured according to standard methods by the same trained physician in each center, as previously detailed [10]. Body mass index (BMI) was calculated as the ratio of weight (Kg) and height ${\mathrm{(meters)}}^{2}$, and transformed into BMI-z score according to the Italian growth charts. Waist circumference was measured in standing position using a flexible tape taken midway between the tenth rib and the iliac crest. The waist-to-height ratio (WHtR) was calculated as the ratio of waist (cm) and height (cm). Blood pressure (BP) was measured in a quiet room and in a seated position using aneroid sphygmomanometer with cuffs of appropriate size, following standard procedures [13]. After 5 min of resting, three measurements were taken, 2 min apart and the mean of the last two values was used in the analyses [13].

After an overnight fast, blood samples were taken for measurement of glucose, insulin and lipids. Glycosylated hemoglobin A1c (HbA1c) was measured by high-performance liquid chromatography in each center [10]. Homeostasis model assessment of insulin-resistance (HOMA-IR) was calculated to estimate insulin-resistance using the following formula: insulin (µU/mL) × fasting glucose (mg/dL)/405. The triglycerides to high-density lipoprotein-cholesterol (TG/HDL-C) ratio was calculated. All young people within the main sample underwent a standard 2-h oral glucose tolerance test (1.75 g of glucose solution per kilogram of body weight, maximum 75 g), and samples were drawn for both glucose and insulin determinations.

Serum creatinine (mg/dL) was measured by kinetic colorimetric Jaffé method in 293 youths and by enzymatic method in 695 youths. Estimated glomerular filtration rate (eGFR) was calculated using Full Age Spectrum for height equation (${\mathrm{eGFRFAS}}_{\text{height}}$): 107.3/(Creatinine/$Q_{\text{height}}$), where Creatinine is expressed in mg/dL and height in meters for both sex. $Q_{\text{height}}$ was calculated as it follows: 3.94 – 13.4 × height + 17.6 × ${\mathrm{height}}^{2}$ – 9.84 × ${\mathrm{height}}^{3}$ + 2.04 × height${\mathrm{height}}^{4}$ [14].

Biochemical tests were performed in the centralized laboratory of each center [10]. Each laboratory belongs to the National Health System and is certified according to International Standards ISO 9000 (http://www.iso9000.it/).

### 2.2 Instrumental Assessments

Liver ultrasonography was performed by experienced radiologists in each center. The presence of FLD was based on the increased echogenicity (brightness) of the liver as compared to the renal cortex [15].

The echocardiographic data were collected with young people in the left lateral decubitus position, using a commercially available echocardiographic system with tissue Doppler (TD) capabilities (Power Vision 8000, Toshiba-Corp. Medical, Japan) equipped with variable frequency phased-array transducer (2.5–3.8 MHz), as elsewhere described [11].

The left ventricular mass (LVM) was calculated according to the American Society of Echocardiography recommendations using M-mode whenever possible, or optimally oriented 2-dimensional parasternal long-axis view. LVM (g) was indexed (LVMi) using a simplified method proposed by Chinali *et al*. [16] and expressed as LVM/[(${\mathrm{height}}^{2.16}$) + 0.09]. Relative wall thickness (RWT) was calculated from the posterior wall thickness, interventricular septum thickness, and left ventricular diastolic diameter through the following formula: (posterior wall thickness + interventricular septum thickness)/left ventricular diastolic diameter. The RWT was normalized for age (${\mathrm{RWT}}_{\text{a}}$) by the following equation: ${\mathrm{RWT}}_{\text{a}}$ = RWT – 0.005 × (age – 10) [17].

Carotid intima media thickness (cIMT) was assessed by commercially available system equipped with a 7–13 MHz linear array probe was used for B-mode ultrasound evaluations. Quantitative B-mode ultrasound measurements of cIMT were obtained as mean of cIMT of near and far walls of both common carotid arteries of both carotid bulbs, as previously described [18].

### 2.3 Definitions

OW or OB were defined using the Italian growth charts [19]. Visceral adiposity was defined as WhtR ≥0.60 [20]. Phenotypes of prediabetes were defined according to the American Diabetes Association i.e.,: impaired fasting glucose (fasting glucose ≥100 mg/dL), impaired glucose tolerance (two-hour glucose during oral glucose tolerance test ≥140 mg/dL), high HbA1c (HbA1c ≥5.7%) [21]. Dyslipidemia was defined using fixed cut-offs proposed by the Expert Panel for Cholesterol (≥200 mg/dL), HDL-Cholesterol (<40 mg/dL), and triglycerides (≥130 mg/dL) [22].

Hypertension was defined by criteria proposed by the American Academy of Pediatrics based of BP ≥95th percentile for age, sex and height in young people aged <13 years and BP ≥130/80 mmHg for adolescents aged ≥13 years [23].

Fatty liver disease was defined as the presence of ultrasound detected hepatic steatosis and assessed as present or absent [12].

Mild reduced eGFR (MReGFR) was defined by a value of ${\mathrm{eGFRFAS}}_{\text{height}}$< 90 ≥ 60 mL/min/1.73 $m^{2}$ [24].

Left ventricular hypertrophy (LVH) was defined using the single cut-point ≥45 ${\mathrm{g/h}}^{2.16}$ [13]. High ${\mathrm{RWT}}_{\text{a}}$ was defined by a cut-off ≥0.38 mm [10]. Concentric LVH was defined by LVH plus high ${\mathrm{RWT}}_{\text{a}}$ [10]. High cIMT was defined by 90th percentile for age and sex by Doyon *et al*. [25].

### 2.4 Statistical Analysis

Variables with normal distribution were expressed as mean ± standard deviation, whereas variables with skewed distribution were Log transformed but expressed as median and interquartile range. Comparison between groups was assessed by Student’s *t* test. Categorical variables were expressed as number and frequence (%) and compared by χ^2^ test. Multiple regression analyses were performed to identify factors associated to LVMi, ${\mathrm{RWT}}_{\text{a}}$ and cIMT. Significance was considered at the level of *p* < 0.05. Statistical analyses were conducted using IBM SPSS Statistics software, Version 28.0 (IBM, Armonk, NY, USA).

## 3. Results

### 3.1 Study Population

The characteristics of the main sample are shown in Table 1. Despite similar age and BMI, boys exhibited higher waist circumference and WHtR than girls (*p* < 0.0001). No sex-related differences were found regarding biochemical or clinical variables, except for higher levels of fasting glucose (*p* = 0.002) in boys, and lower eGFRF in girls (*p* < 0.0001).

**Table 1.** **Description of the main sample as a whole and by sex**.

	All	Boys	Girls	*p* value
*n*	988	510	478	
Age, years	12.9 ± 1.8	12.8 ± 1.7	13.0 ± 1.9	0.063
BMI (${\mathrm{kg/m}}^{2}$)	32.1 ± 5.4	32.0 ± 5.5	32.2 ± 5.4	0.607
BMI-z score	2.39 ± 0.63	2.36 ± 0.64	2.42 ± 0.62	0.183
Waist circumference (cm)	97.0 ± 12.7	98.9 ± 12.9	95.0 ± 12.3	<0.0001
Waist-to-height ratio	0.616 ± 0.08	0.623 ± 0.08	0.609 ± 0.08	<0.0001
$G_{0}$ (mg/dL)	88.6 ± 9.7	89.5 ± 9.4	87.6 ± 9.9	0.002
$G_{120}$ (mg/dL)	111.5 ± 21.2	112.3 ± 19.4	110.7 ± 22.8	0.226
HbA1c (%)	5.3 ± 0.4	5.3 ± 0.4	5.3 ± 0.4	0.169
HOMA-IR	4.0 (2.7–6.0)	3.9 (2.7–5.6)	4.2 (2.7–6.2)	0.221
Cholesterol (mg/dL)	153.9 ± 28.7	152.6 ± 28.2	155.4 ± 29.3	0.130
HDL-C (mg/dL)	47.0 ± 10.1	47.2 ± 9.8	46.8 ± 10.4	0.524
Triglycerides (mg/dL)	80.5 (62.0–105.0)	78.0 (60.8–102.0)	84.5 (63.8–109.3)	0.054
TG/HDL-C ratio	1.8 (1.3–2.5)	1.7 (1.2–2.4)	1.9 (1.3–2.5)	0.051
Systolic BP (mmHg)	116.1 ± 13.0	116.7 ± 12.8	115.4 ± 13.2	0.105
Diastolic BP (mmHg)	69.2 ± 9.4	69.0 ± 9.3	69.4 ± 9.4	0.425
eGFR (mL/min/1.73 $m^{2}$)	114.3 ± 22.4	117.1 ± 21.9	111.3 ± 22.5	<0.0001

Data are expressed as mean ± standard deviation, median (interquartile range), n (%). BMI, body mass index; BP, blood pressure; eGFR, estimated glomerular filtration rate; $G_{0}$, fasting glucose; $G_{120}$, glucose at 120’ during oral glucose tolerance test; HbA1c, glycosylated hemoglobin A1c; HOMA-IR, homeostasis model assessment of insulin-resistance; TG/HDL-C, triglycerides to high-density lipoprotein-cholesterol.

**Fig. 1.**
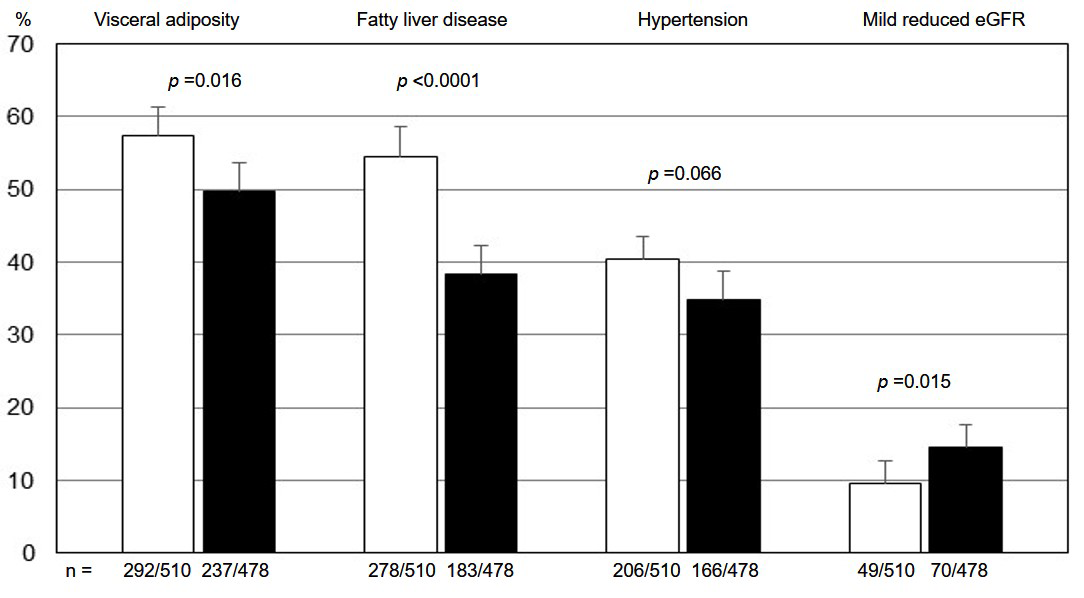
**Proportion (95% Cl) of youths with visceral adiposity, fatty liver disease, hypertension, and mild reduced estimated glomerular filtration rate in boys (white bars) and girls (black bars)**. eGFR, estimated glomerular filtration rate.

When the categorical variables were considered, more boys than girls showed a WhtR ≥0.60 (*p* = 0.016), FLD (*p* < 0.0001) (Fig. 1) and hypertension, although at a lesser extent (*p* = 0.066). On the contrary, a higher frequence of MReGFR was observed in girls (*p* = 0.015). No differences were observed regarding phenotypes of prediabetes or dyslipidemia (**Supplementary Fig. 1**). Impaired glucose tolerance was slightly more frequent in girls than boys: 10.7% vs 7.5% (*p* = 0.077).

### 3.2 Left Ventricular Echocardiography

The main characteristics of the sample who underwent echocardiographic evaluation are reported in Table 2.

**Table 2.** **Description of the sample with echocardiographic evaluation as a whole and by sex**.

	All	Boys	Girls	*p* value
*n*	142	67	75	
Age, years	12.2 ± 1.8	11.9 ± 1.5	12.4 ± 2.0	0.068
BMI (${\mathrm{kg/m}}^{2}$)	29.8 ± 4.8	29.5 ± 5.1	30.1 ± 4.4	0.493
BMI-z score	2.1 ± 0.6	2.1 ± 0.6	2.2 ± 0.6	0.300
Waist-to-height ratio	0.623 ± 0.07	0.626 ± 0.07	0.620 ± 0.06	0.545
Cholesterol (mg/dL)	162.5 ± 35.2	157.7 ± 34.2	166.8 ± 35.7	0.123
HDL-C (mg/dL)	48.8 ± 11.6	47.6 ± 11.4	49.9 ± 11.7	0.239
Triglycerides (mg/dL)	87.0 (59.0–107.0)	86.0 (55.0–99.0)	88.0 (63.0–120.0)	0.115
TG/HDL-C ratio	1.8 (1.2–2.5)	1.8 (1.0–2.4)	1.7 (1.2–2.5)	0.433
Systolic BP (mmHg)	109.7 ± 10.9	110.2 ± 11.5	109.3 ± 10.4	0.603
Diastolic BP (mmHg)	66.5 ± 10.0	68.2 ± 9.5	65.0 ± 10.8	0.009
LVMi (${\mathrm{g/m}}^{2.16}$)	44.2 ± 12.1	46.3 ± 10.9	42.3 ± 12.8	0.046
${\mathrm{RWT}}_{\text{a}}$	0.351 ± 0.06	0.367 ± 0.06	0.337 ± 0.06	0.003

Data are expressed as mean ± standard deviation, median (interquartile range), n (%). BMI, body mass index; BP, blood pressure; HDL-C, high-density lipoprotein cholesterol; TG/HDL-C, triglycerides to high-density ipoprotein-cholesterol; LVMi, left ventricular mass index; ${\mathrm{RWT}}_{\text{a}}$, relative wall thickness for age.

Despite no sex-related differences were observed for age and BMI, boys exhibited higher values of LVMi (*p* = 0.046) and ${\mathrm{RWT}}_{\text{a}}$ (*p* = 0.003) as compared to girls.

Boys exhibited a higher frequence of LVH (*p* = 0.015) or concentric LVH (*p* = 0.002) (**Supplementary Fig. 2**). Multiple regression analysis showed that LVMi was significantly and independently associated with WHtR, TG/HDL-C ratio and and male sex (**Supplementary Table 1**). ${\mathrm{RWT}}_{\text{a}}$ was significantly associated with WHtR and male sex.

### 3.3 Carotid Intima Media Thickness

The features of youths who underwent a cIMT evaluation are reported in Table 3. Boys showed similar age and BMI compared to girls, but they presented higher values of cIMT (*p* = 0.011). cIMT values >90th percentile for age and sex were found in 76% of the total sample (boys 86.4% and girls 62.5%, *p* = 0.004). cIMT was independently associated only with age and male sex (**Supplementary Table 1**).

**Table 3.** **Description of the sample with evaluation of carotid intima-media thickness as a whole and by sex**.

	All	Boys	Girls	*p* value
*n*	107	59	48	
Age, years	12.1 ± 1.5	12.3 ± 1.4	11.9 ± 1.5	0.220
BMI (${\mathrm{kg/m}}^{2}$)	31.5 ± 5.5	31.3 ± 5.1	31.6 ± 6.1	0.756
BMI-z score	2.3 ± 0.6	2.3 ± 0.6	2.4 ± 0.7	0.484
Waist-to-height ratio	0.653 ± 0.07	0.652 ± 0.07	0.650 ± 0.08	0.856
Cholesterol (mg/dL)	154.6 ± 32.4	149.5 ± 30.5	161.2 ± 33.9	0.068
HDL-C (mg/dL)	44.9 ± 10.3	44.5 ± 11.3	45.4 ± 8.9	0.628
Triglycerides (mg/dL)	86.0 (68.0–115.0)	86.0 (64.5–109.8)	87.5 (68.0–119.0)	0.459
TG/HDL-C ratio	1.9 (1.4–2.8)	2.1 (1.4–2.7)	1.9 (1.5–3.0)	0.695
Systolic BP (mmHg)	122.2 ± 13.2	122.1 ± 13.5	122.4 ± 13.0	0.886
Diastolic BP (mmHg)	79.1 ± 8.3	79.2 ± 8.0	79.0 ± 8.7	0.896
cIMT (mm)	0.50 ± 0.06	0.52 ± 0.06	0.49 ± 0.07	0.011

Data are expressed as mean ± standard deviation, median (interquartile range), n (%). BMI, body mass index; BP, blood pressure; cIMT, carotid intima media thickness; TG/HDL-C, triglycerides to high-density lipoprotein-cholesterol.

## 4. Discussion

The present study highlighted several sex-related differences in cardiovascular risk factors in adolescents with OW or OB, demonstrating that boys presented a higher degree of visceral adiposity, FLD, cardiac and vascular abnormalities compared to girls, while girls showed a higher risk of mild reduced glomerular function.

These findings are consistent with the sex-related cardiovascular risk described in non-elderly adults. Indeed, it is well established that males are considered at higher risk of cardiovascular morbidity and mortality than females, although this difference varies over time and geographically [26]. Due to the multifactorial nature of cardiometabolic risk, sex-related differences can be explained not only by the protective role of estrogens, but also by the complex interplay of several conditions, such as those correlated to lifestyle behaviours (e.g., smoking, alcohol consumption), or traditional cardiometabolic risk factors (such as visceral adiposity, dyslipidemia, and hypertension) which are more prevalent in non-elderly and non-diabetic males than females [26].

More complex and conflicting data are available on the role of sex as non modifiable risk factor for cardiovascular mortality among adults with OW or OB. For instance, the hazard ratio of cardiovascular death was higher in men with obesity (BMI ≥ 30 < 40 ${\mathrm{kg/m}}^{2}$) [1.55 (1.22–1.96)] than in females [1.09 (0.85–1.40)] as demonstrated by Khan *et al*. [27] by analyzing several longitudinal studies in the United States (Cardiovascular Disease Lifetime Risk Pooling Project). Another study demonstrated that men showed a higher mortality for cardiovascular disease as compared to women independently of the severity of OB on the basis of 11 prospective cohorts from four European countries [28]. On the contrary, Mongraw-Chaffin *et al*. [29] reported that higher BMI had the same deleterious effect on risk of incident cardiac heart disease in women and men.

Robust evidence has linked traditional pediatric cardiometabolic risk factors, such as increased adiposity, hypertension, hyperlipidemia, and risk factor clustering with subclinical cardiovascular disease [30]. The adverse cardiometabolic risk profile related to OB starting in early adolescence and even in early childhood has been also associated with fatal and not fatal cardiovascular events as early as 40 years of age [31].

The association between OB and cardiometabolic risk factors may begin at different stages of youth, depending on the degree of OB, cardiometabolic risk factors, and sex [32]. The emergence of sex-related differences in the trajectories of atherogenic lipids (apolipoprotein B containing very low-density lipoprotein and low density lipoprotein traits) and predictive biomarkers (glucose and HDL-C) for cardiometabolic diseases has been demonstrated in a prospective birth cohort study from childhood (age 7 years) to early adulthood (25 years) in United Kingdom [33]. Most changes of causal and predictive cardiometabolic traits were detrimental for males, and emerged only in late childhood and adolescence. It may be possible that multiple mediators such as adiposity, puberty timing, and other health behaviours might have played a role, but this aspect was not assessed in that study.

Of note, we did not find any sex-related difference in the traditional cardiometabolic risk factors, either when they were considered as continuous or categorical variables, in our sample of adolescents with OW or OB, except for higher values of fasting plasma glucose in boys and a slightly higher frequence of impaired glucose tolerance in girls. Moreover, a significally higher WHtR values and frequence of individuals with WHtR ≥0.60 were found in boys. It is well known the important role played by visceral fat on cardiovascular risk. In particular, the WHtR is a sex- and age-independent proxy for visceral adiposity and it is strongly associated with higher cardiometabolic risk in children with OW or OB. Fundamentally, our data are in partial agreement with another cross-sectional study of treatment-seeking Norwegean adolescents with severe obesity, in whom no sex-related differences were found in diastolic BP, total cholesterol, HOMA-IR, and HbA1c levels [34]. Instead, several discordances were noted likely due to the different genetic, environmental or socio-economic background. Firstly, we did not confirm the higher systolic BP values and the lower HDL-Cholesterol values reported in boys in the Norwegean study. Secondly, we found that girls and not boys were exposed to higher levels of triglycerides and had a higher TG/HDL-C ratio, whereas boys had higher levels of fasting glucose that were non reported in the Norwegean study.

Similarly to visceral adiposity, we found a higher frequence of FLD in boys than girls. The close relationship of FLD with cardiometabolic risk in children with OB has been largely demonstrated [34, 35, 36]. As observed in adults [37, 38, 39], accumulating pediatric evidence described non-alcoholic FLD as a condition with sexual dimorphism, with a higher prevalence in boys than in girls [40, 41, 42]. This might be attributable to the predominant visceral distribution of adipose tissue in males that is associated with insulin resistance and free fatty acids flux, leading to FLD development [39]. On the other hand, females have a prevalent subcutaneous fat distribution and leptin production that prevents from visceral fatty tissue accumulation in cooperation with estrogens, which may play a protective role against liver fat accumulation. However, the role of sex steroids on metabolic impairments is still complex and needs to be further elucidated [41, 42, 43].

In the wide perspective of cardiometabolic burden of pediatric obesity, the obesity-related glomerulopathy has recently gained remarkable attention [44, 45]. Similarly to adults [46], emerging data demonstrated that children with OB are at higher risk of kidney damage (expressed as renal function decline with or without hypertension and/or proteinuria) [47, 48, 49, 50]. Of note, cardiometabolic parameters have been closely associated to kidney injury, suggesting an intimate link between renal function and OB, but also with the obesity-related dysmetabolic state [45, 51, 52].

In line with adult evidence reporting that females are more susceptible to chronic kidney disease development than males [53, 54], we demonstrated for the first time sex-related differences for mild renal injury in adolescents with OB.

It is well established that LVH or abnormal LV geometry act as risk factors for cardiovascular morbidity and mortality in adult populations [55, 56]. The assessment of LVH depends on the cut-off used to calculate LV mass or on the presence of hypertension, which is the principal determinant of LVH [13]. In any case, using the method proposed by Chinali *et al*. [16], that indexes the LVM for ${\mathrm{height}}^{2.16}$ with a single cut-point for both sex, we observed higher LVMi, and higher prevalence of LVH and even of concentric LVH in boys than in girls, as demonstrated in adults. Of note, we observed lower levels of ${\mathrm{RWT}}_{\text{a}}$ in girls vs boys, likely due to several factors such as body weight or fat mass. This finding may be expression of reduced adaptation of LVM to increased body weight or to the postload in girls vs boys. This kind of cardiac geometry may predispose later in life to higher risk of eccentric LVH notoriously associated with cardiac failure that is prevalent in females than males with OB [57].

With regard to cIMT as a marker of preclinical atherosclerosis in adolescents, studies regarding the impact of sex are limited and not conclusive. In addition, the lack of robust normative tables makes difficoult the interpretation of cIMT [56]. We observed a higher prevalence of high cIMT in boys than girls, but the finding that approximately 76% of youths with OW/OB had cIMT levels above 90th percentile [25] highlights the difficulty of interpreting cIMT in adolescents with OW or OB from a clinical point of view.

This study has some limitations that should be acknowledged. Firstly, the cross sectional design does not allow to understand whether the sex-related differences in the traditional cardiometabolic risk factors might increase in the following years. Secondly, information about pubertal stage, family history or lifestyle behaviours were not available to assess their possible influence on the sex prevalence of visceral adiposity, hypertension, fatty liver disease, and preclinical signs of target organ damage. On the other hand, study strengths include the multicenter study design and the large and well-phenotyped sample allowing to show significant sex-differences in the cardiovascularrisk profile of children with obesity.

## 5. Conclusions

In conclusion, this cross-sectional study conducted in adolescents with OW/OB confirmed our hypothesis that sex-related differences in cardiovascular risk might be present in adolescents with OW/OB. Boys exhibited a higher degree of visceral adiposity, hypertension, FLD, LVH, and cIMT than girls. Conversely, a higher prevalence of MReGFR was detectable in girls. These findings mirror the cardiovascular risk profile observed in non-elederly and non-diabetic adults. Considering that cardiovascular risk might occur early in life, the role of a non modifiable factor such as sex should be considered for early cardiovascular risk stratification.

Worthy of note, relevant clinical and prognostic implications could be also drawn. Indeed, a better understanding of sex-differences related to cardiometabolic health of children with OW/OB might enhance the effectiveness of obesity prevention interventions by significantly improving the challenging management of these at-risk patients in clinical practice.

Therefore, more research efforts are needed to expand knowledge about sex-differences in the context of pediatric OB. As a matter of fact, this could pave the way for an insightful approach of personalized medicine through targeted strategies for children with OB.

## Data Availability

The datasets used and analyzed during the current study are available from the corresponding author on reasonable request.

## Abbreviations

BP, blood pressure; BMI, body mass index; cIMT, carotid intima media thickness; ${\mathrm{eGFRFAS}}_{\text{height}}$, Full Age Spectrum for height equation; FLD, fatty liver disease; GFR, glomerular filtration rate; HDL-C, high-density lipoprotein cholesterol; HbA1c, glycosylated hemoglobin A1c; HOMA-IR, Homeostasis model assessment of insulin-resistance; IVST, interventricular septum thickness; LVH, left ventricular hypertrophy; LVM, left ventricular mass; MReGFR, mild reduced eGFR; OB, obesity; OW, overweight; RWT, relative wall thickness; TG/HDL-C ratio, triglycerides to high-density lipoprotein-cholesterol ratio; WHtR, waist to height ratio.

## Supplementary Material

Supplementary material associated with this article can be found, in the online version, at https://doi.org/10.31083/j.rcm2504141.
