# Supplementary material for: Sex-Related Differences in Cardiovascular Risk in Adolescents with Overweight or Obesity
Source: Rev Cardiovasc Med. 2024 Apr 9;25(4):141. doi: 10.31083/j.rcm2504141 (PMC11264036; doi:10.31083/j.rcm2504141)
Supplement: Supplementary file 1 [file 2153-8174-25-4-141-s1.docx]

**Supplementary material**

**Supplementary Fig. 1. Prevalence of phenotypes of prediabetes and dyslipidemia in boys (grey bars) and girls (black bars).**


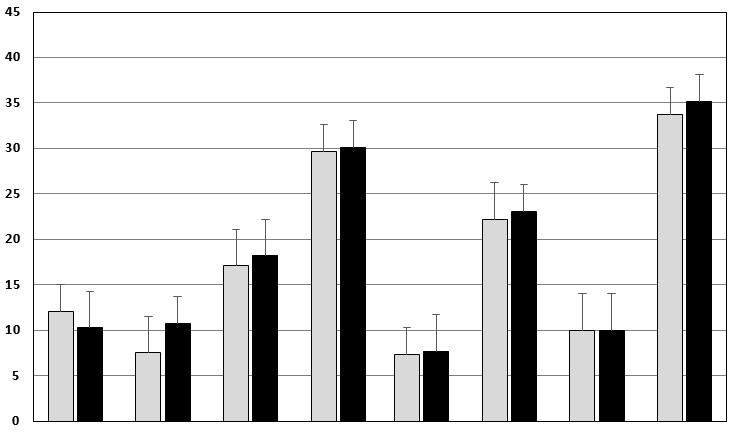


% IFG IGT High HbA1c Prediabetes High TC Low HDL-C High TG Dyslipidemia

*p* =0.393

*p* =0.077

*p* =0.638

*p* =0.859

*p* =0.772

*p* =0.748

*p* =0.983

*p* =0.638

**IFGI** BP, Blood pressure; BMI , Body mass index; cIMT, carotid intima media thickness; ~~CKD, chronic kidney disease;~~ ~~CMRF, cardiometabolic risk factors;~~ ~~CVD, cardiovascular disease~~, ~~CVR, cardiovascular risk~~; ~~CVRFs, CVR factors;~~ eGFRFAS_height,_ Full Age Spectrum for height equation; FLD,fatty liver disease; GFR, glomerular filtration rate; HDL-C, high-density lipoprotein cholesterol; HOMA-IR, Homeostasis model assessment of insulin-resistance; ~~IFG , impaired fasting glucose;~~ ~~IGT, impaired glucose tolerance~~; ~~ISPED, Italian Society for Pediatric Endocrinology and Diabetology~~; IVST, interventricular septum thickness; ~~LDL, low-density lipoprotein~~; ~~LVDD, left ventricular mass LV diastolic diameter,~~ LVM, left ventricular mass; MReGFR, mild reduced eGFR; ~~NAFLD, non-alcoholic fatty liver disease; OGTT, oral glucose tolerance test~~; OB, obesity; OW, overweight; ~~PWT, posterior wall thickness~~; RWT, relative wall thickness; SDS, standard deviation score; ~~TOD, target organ damage~~; TG/HDL-C ratio, triglycerides to high-density lipoprotein-cholesterol ratio; ~~VLDL , very low-density lipoprotein~~; ~~WC, Waist circumference~~; WhtR, waist to height ratio.

IFG: impaired fasting glucose; IGT: impaired glucose tolerance; HbA1c: glycosylated haemoglobin; HDL-C: high-density lipoprotein-cholesterol; TC: total cholesterol; TG: triglycerides.

**Supplementary Fig. 2. Proportion of youths with left ventricular hypertrophy or concentric left ventricular hypertrophy in boys (greys bars) and girls (black bars).**

% Left ventricular hypertrophy Concentric left ventricular hypertrophy

**
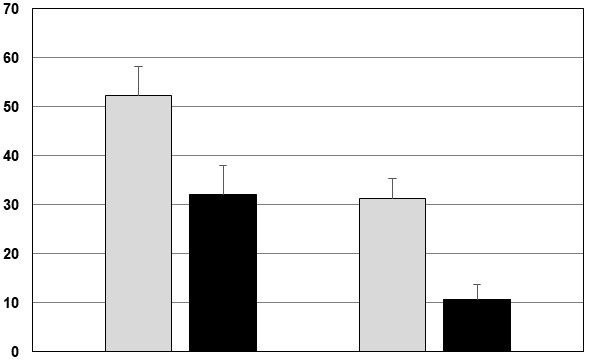
**

*p* =0.002

*p* =0.015

n= 35/67 24/75 21/67 8/75

**Supplementary Table 1. Factors independently associated with left ventricular mass index and relative wall thickness or carotid intima media thickness by multiple regression analysis.**

|  | B coefficient | SE | T | *p* |
| --- | --- | --- | --- | --- |
| LVM/height^2.16^ (g/m^2.16^) |  |  |  |  |
| WHtR | 50.00 | 15.5 | 3.3 | 0.001 |
| TG/HDL ratio | 10.0 | 4.1 | 2.4 | 0.017 |
| Boys | 4.6 | 1.9 | 2.4 | 0.017 |
| RWT_a_ |  |  |  |  |
| WHtR | 0.17 | 0.08 | 2.3 | 0.022 |
| Boys | 0.029 | 0.01 | 2.9 | 0.004 |
| cIMT (mm) |  |  |  |  |
| Age | 0.011 | 0.004 | 2.8 | 0.006 |
| Boys | 0.024 | 0.12 | 2.0 | 0.044 |

cIMT: carotid intima media thickness; LVM: left ventricular mass; RWT_a_, relative wall thickness normalized for age; TG/HDL-C: triglycerides to high-density lipoprotein-cholesterol ratio; WHtR: waist-to-height ratio.
